# Role of Apolipoprotein E in the Hippocampus and Its Impact following Ionizing Radiation Exposure

**DOI:** 10.3390/cells13110899

**Published:** 2024-05-23

**Authors:** Arianna Casciati, Emanuela Pasquali, Ilaria De Stefano, Ignacia Braga-Tanaka, Satoshi Tanaka, Mariateresa Mancuso, Francesca Antonelli, Simonetta Pazzaglia

**Affiliations:** 1 Division Health Protection Technologies, Italian National Agency for New Technologies, Energy and Sustainable Economic Development (ENEA), 00123 Rome, Italy; arianna.casciati@enea.it (A.C.); emanuela.pasquali@enea.it (E.P.); ilaria.destefano@enea.it (I.D.S.); mariateresa.mancuso@enea.it (M.M.); 2 Department of Radiobiology, Institute for Environmental Sciences, Rokkasho 039-3212, Japan; tanakaib@ies.or.jp (I.B.-T.); tanakas@ies.or.jp (S.T.)

**Keywords:** hippocampus, adult neurogenesis, ApoE mice, radiation, dose-rate effects

## Abstract

Apolipoprotein E (ApoE) is a lipid carrier in both the peripheral and the central nervous systems (CNSs). Lipid-loaded ApoE lipoprotein particles bind to several cell surface receptors to support membrane homeostasis and brain injury repair. In the brain, ApoE is produced predominantly by astrocytes, but it is also abundantly expressed in most neurons of the CNS. In this study, we addressed the role of ApoE in the hippocampus in mice, focusing on its role in response to radiation injury. To this aim, 8-week-old, wild-type, and ApoE-deficient (ApoE^−/−^) female mice were acutely whole-body irradiated with 3 Gy of X-rays (0.89 Gy/min), then sacrificed 150 days post-irradiation. In addition, age-matching ApoE^−/−^ females were chronically whole-body irradiated (20 mGy/d, cumulative dose of 3 Gy) for 150 days at the low dose-rate facility at the Institute of Environmental Sciences (IES), Rokkasho, Japan. To seek for ApoE-dependent modification during lineage progression from neural stem cells to neurons, we have evaluated the cellular composition of the dentate gyrus in unexposed and irradiated mice using stage-specific markers of adult neurogenesis. Our findings indicate that ApoE genetic inactivation markedly perturbs adult hippocampal neurogenesis in unexposed and irradiated mice. The effect of ApoE inactivation on the expression of a panel of miRNAs with an established role in hippocampal neurogenesis, as well as its transcriptional consequences in their target genes regulating neurogenic program, have also been analyzed. Our data show that the absence of ApoE^−/−^ also influences synaptic functionality and integration by interfering with the regulation of mir-34a, mir-29b, and mir-128b, leading to the downregulation of synaptic markers PSD95 and synaptophysin mRNA. Finally, compared to acute irradiation, chronic exposure of ApoE null mice yields fewer consequences except for the increased microglia-mediated neuroinflammation. Exploring the function of ApoE in the hippocampus could have implications for developing therapeutic approaches to alleviate radiation-induced brain injury.

## 1. Introduction

High-dose cranial radiation therapy is linked to various adverse effects on the central nervous system (CNS), including cognitive function decline [1]. Even lower radiation doses can result in impairments in cognitive function without apparent structural changes [2]. In fact, the ICRP 2012 raised attention to circulatory disease at low doses, because of recent evidence of increased risks not only at doses above 5 Gy but also in a range of doses from 5 to 0.5 Gy [3]. The underlying mechanisms of radiation-induced cognitive injury, particularly at lower doses and dose-rates, are still not fully understood. In rodent studies, ionizing radiation hampers adult neurogenesis by eliminating neural precursor cells in the subgranular zone (SGZ) of the hippocampus, with the extent of damage correlating with the radiation dose.

Apolipoprotein E (ApoE) is a lipid carrier, and it is the most important apolipoprotein in the brain tissue. Lipid-loaded ApoE lipoprotein particles bind to several cell surface receptors to support membrane homeostasis and injury repair in the brain. In the brain, ApoE is produced predominantly by astrocytes, activated microglia, vascular mural cells, choroid plexus cells, and, to some extent, by stressed neurons in response to excitotoxic injury [4].

Three ApoE isoforms exist in humans, ApoE2, ApoE3, and ApoE4, encoded by three different alleles, *ApoE ε*2, *ε*3 and *ε*4, respectively, differing by the arginine/cysteine residues at positions 112 and 158, causing structural and functional differences between these isoforms [5]. Noteworthy, the ε4 allele of *ApoE*, which is present in 15–20% of the population, represents the primary risk factor for late-onset Alzheimer Disease (AD), with a prevalence of 50–80% in AD patients. ApoE4 carriers have increased amyloid deposition due to impeded Aβ clearance, for all the forms, with ApoE4 having the strongest effect. Therefore, lowering ApoE holds promise in therapy. Indeed, it has been reported that haploinsufficiency of ApoE3/ApoE4 in an Aβ-overproducing mouse model reduced Aβ levels [6], and treatment with anti-ApoE antibody decreases amyloid deposition, also improving cognition in an AD mouse model [7].

In other species, including mice, *ApoE* exists only in one form [8]. *ApoE* has been implicated in hippocampal dentate gyrus (DG) development, where its absence has been shown to inhibit neurogenesis [9]. Importantly, *ApoE*-deficient mice show decreased locomotor activity in novel environments and learning and memory deficits, consistent with cognitive impairment and memory loss [10]. Beside learning deficits, *ApoE*-deficient mice also exhibit age-dependent synaptic loss and blood–brain barrier (BBB) breakdown [11], especially after traumatic brain injury [12], suggesting *ApoE* requirement for a healthy brain aging [13]. These discrepancies on whether ApoE lowering is therapeutic or is necessary for healthy brain function require further investigations on the role of *ApoE* in homeostasis and brain repair.

Since ApoE has been shown to be required in the DG for recovery after injury [14,15], we have conducted in vivo experiments using the *ApoE* KO mice to investigate the role of ApoE on adult hippocampal neurogenesis and how exposure to ionizing radiation affects hippocampal neurogenesis when ApoE is lacking. As behavioral results show gender-related differences, with *ApoE* KO female mice displaying greater cognitive impairment [16], only females were employed here to avoid confounding factors. Furthermore, we investigate the dose-rate effects by exposing *ApoE* KO mice to radiation in the low dose-rate facility at the Institute of Environmental Sciences (IES), Rokkasho, Japan. In our study, we compared the effects induced by a cumulative dose of 3 Gy of low LET radiation administered either chronically (γ-rays, 20 mGy/day for 150 days) or acutely (X-rays, dose rate of 0.89 Gy/min). The effect of *ApoE* inactivation on hippocampal astroglia and microglia was also investigated, as well as its transcriptional consequences in genes regulating neurogenic program. Since cell-intrinsic gene expression programs are also controlled by cell-extrinsic signals such as miRNAs, the expression levels of a specific group of miRNAs known to be involved in hippocampal neurogenesis and synaptic function were examined in the hippocampi of untreated and irradiated wild-type (WT) and *ApoE^−/−^* mice, to test the possible involvement of miRNA in the alteration of adult neurogenesis dependent on ApoE deficiency.

## 2. Materials and Methods

### 2.1. Animals

*ApoE* knockout (*ApoE^−/−^*) female mice on C57BL/6J background (Charles River Laboratories, Calco, Italy) were bred in the animal facility at ENEA under conventional conditions, or at the IES animal facility at Rokkasho, Japan under specific pathogen-free (SPF) conditions. 

All experiments were conducted in compliance with the Directive 2010/63/EU of the European Parliament, approved by the local Ethical Committee for Animal Experiments of the ENEA, and authorized by the Italian Ministry of Health (n°132/2015-PR). Additionally, experiments in Japan adhered to the relevant legal regulations in that country. At both animal facilities, the mice were provided with standard rodent chow *ad libitum* and kept under a 12 h light/dark cycle for maintenance.

### 2.2. Irradiation

*ApoE^−/−^* female mice of 8 weeks of age were acutely or chronically irradiated. Acute irradiation with a single dose of 3 Gy of X-rays was performed at ENEA using a Gilardoni CHF 320 G X-ray generator (Gilardoni, Mandello del Lario, Italy) operated at 250 kVp, 15 mA, with Half-Value Layer = 1.6 mm Cu (additional filtration of 2.0 mm Al and 0.5 mm Cu), at a dose rate of 0.89 Gy/min [17]. For low dose-rate exposures, mice were chronically irradiated at IES with γ-rays from a ^137^Cs source (22 h/day), reaching a cumulative total dose of 3 Gy in 150 days (dose rate of 20 mGy/day). 

Acutely irradiated mice were euthanized by CO_2_ asphyxiation 150 days after exposure. Chronically irradiated mice were analogously euthanized at the same age (Table 1). Controls were sham-irradiated. Brains were collected from sham-irradiated and irradiated mice for histological and molecular analysis.

### 2.3. Histological Analysis

Mouse brains were fixed in 10% neutral buffered formalin, and paraffin embedded according to standard protocols, both at ENEA (n = 6) and IES (n = 6). To assess adult neurogenesis, each mouse brain was sagittally sectioned along the midline, and sections were collected starting approximately 300 μm away from the midline. To ensure consistency in the counting process, cell quantification was conducted on three non-overlapping consecutive sections obtained from both hemispheres of each brain considered for the analysis, representing the rostral/mid hippocampus region. Fixed brain sections were immunostained as described in [18] using the following primary antibodies diluted as indicated by the manufacturer: GFAP (Z0334, Dako, Jena, Germany, 1:500), SOX2 (ab97959, Abcam, Cambridge, UK, 1:500), DCX (18723, Abcam, Cambridge, UK, 1:2000), NeuN (MAB377, Millipore, Darmstadt Germany 1:100), Ki67 (ab15580, Abcam, Cambridge, UK, 1:200), and IBA1 (019-19741, Wako, Richmond, VA, USA, 1:500). The quantification of positive cells in the SGZ was presented as the number of positive cells per millimeter of SGZ length. Radial glia-like cells (RGLs) were identified based on specific criteria, which included their location within the SGZ, positive labeling, and morphology. Immunohistochemical staining for NeuN was conducted within a rectangular field measuring 2000 mm^2^ in both the supra- and infrapyramidal blades and in the crest area of the DG. Quantitative analysis of astroglial cells, identified through labeling with anti-GFAP antibody, was performed in the molecular layer (ML) and in the hilus (H) of the hippocampus and expressed as astrocytes per square micrometer (astrocytes/μm^2^). Images for quantification were taken using the imaging software NIS-Elements BR 4.00.05 (Nikon Instruments Europe B.V., Amsterdam, The Netherlands). All the experiments were analyzed blindly by two independent researchers to minimize bias. Statistical analyses were carried out using GraphPad Prism 6.0 (GraphPad Software, Inc., San Diego, CA, USA) and statistical significance was determined using a two-tailed Student’s *t*-test for comparison between pairs of means. The *p*-values < 0.05 were statistically significant.

### 2.4. Real-Time qPCR

At 7 months of age, hippocampi (n = 3/group) from irradiated (acute and chronic exposure) and age-matched sham-irradiated controls were collected in RNAprotect Tissue Reagent (Qiagen, Hilden, Germany), and DGs were manually dissected under a stereomicroscope. After dissection, DG samples were stored at −80 °C in RNAprotect Tissue Reagent until they were ready for RNA extraction. Three hippocampi for each condition were pulled together and the total RNA and miRNA-enriched fraction were extracted using miRNeasy Tissue/Cells Advanced Micro Kit (Cat. No./ID: 217684; Qiagen, Milan, Italy) according to the manufacturer’s instruction. After extraction, all samples were quantified by a NanoDrop spectrophotometer (Thermo Fisher Scientific Inc., Waltham, MA, USA), and 1 μg of total RNA was reverse transcribed with a High-Capacity cDNA Reverse Transcription Kit (Applied Biosystems, Foster City, CA, USA), then stored at −20 °C until analyzed. Primers were designed for target genes working under the same cycling conditions (Table 2). Real-time quantitative PCR (qPCR) was performed by StepOnePlus Real-Time PCR System (Applied Biosystems) using Power SYBR Green PCR Master Mix (Applied Biosystems). Reactions were performed in triplicate. Relative gene expression was quantified using glyceraldehyde-3-phosphate dehydrogenase (GADPH) as a housekeeping gene.

The standard curve method was used to normalize expression of the reference gene and to calculate the relative expression levels of target genes.

The miRNA analysis was performed using the Applied Biosystems TaqMan Advanced miRNA Assays, enabling highly sensitive and specific quantification of mature miRNAs by qPCR (Thermo Fisher Scientific Waltham, MA, USA). The QIAGEN Primers used were mhu-let-7b-5p (Assay Name 002619), mhu-miR-9a-5p (Assay ID 478214_mir), mhu-miR-34a-5p (Assay ID 478052_mir), mhu-miR-125b-5p (Assay ID 477885_mir), mhu-miR-29b-3p (Assay ID 478369_mir), mhu-miR-128-3p (Assay Id 477892 mir), and U6 snRNA (Assay ID 01973) as housekeeping. The ΔΔCt quantitative method was used to normalize the expression of the reference gene and to calculate the relative expression levels of target genes. The statistical significance was calculated by two-tailed Student’s *t*-test for comparison between pairs of means. The *p*-values < 0.05 were statistically significant.

## 3. Results

### 3.1. Effects of ApoE Deficiency on Adult Hippocampal Neurogenesis and Neuroinflammation

**Figure 1:**
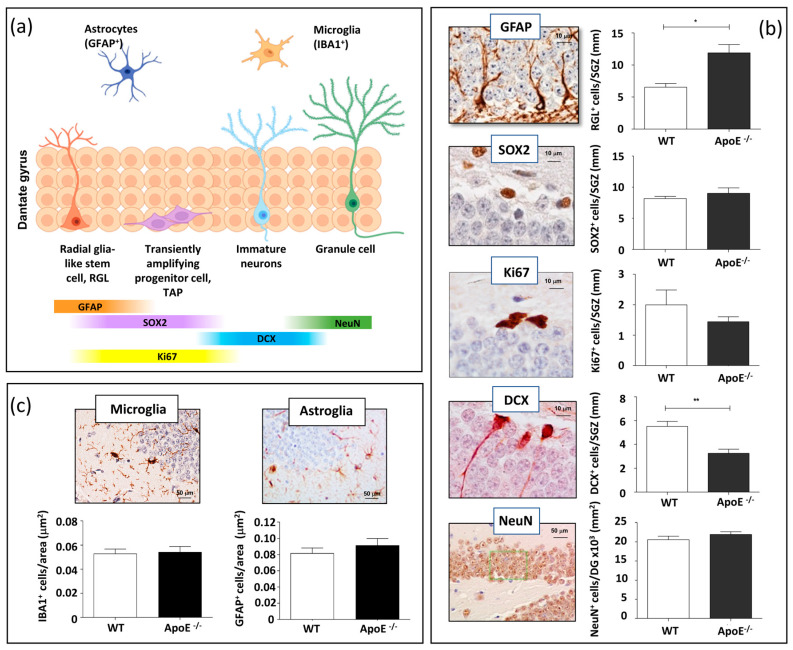
Effects of ApoE deficiency on adult hippocampal neurogenesis and neuroinflammation. (**a**) Scheme of the adult hippocampal neurogenesis process. (**b**) Representative images of stage-dependent neurogenesis markers (GFAP, SOX2, Ki67, DCX and NeuN) and relative quantification in the DG of WT and *ApoE^−/−^* mice. (**c**) Representative images of microglial and astroglial labelling relative quantification of the number of positive cells per μm^2^ of ML/H area in WT and *ApoE^−/−^* mice. Data are shown as mean ± SEM. Differences were tested with Student’s *t*-test. * *p* < 0.05; ** *p* < 0.01.

Adult hippocampal neurogenesis is a multistep process that originates from a sequence of proliferative precursor cells and leads to a new granule cell in the DG. Slowly dividing/quiescent RGLs, featured with a single radial process that extends through the granular cell layer of the DG, express glial fibrillary acid protein (GFAP). RGLs give rise to transit amplifying progenitors (TAPs) that are small round cells expressing SRY (sex determining region Y) box 2 (SOX2), endowed with high proliferative activity (Ki67). The first indications of neuronal lineage choice appear in newborn neurons expressing doublecortin (DCX). Newborn neurons progress into mature granule neurons (NeuN) and finally integrate in the existing neuronal circuit (Figure 1a) [19]. 

To investigate the role of ApoE on adult hippocampal neurogenesis in vivo and to detect possible ApoE-dependent modifications in the cellular composition of the SGZ of the DG, we evaluated stage-specific adult neurogenesis markers in *ApoE^−/−^* and WT mice at 7 months of age, following immunohistochemical analysis based on morphological and labelling characteristics (Figure 1b). To this aim, GFAP, Ki67, SOX2, DCX, and NeuN were evaluated in the DG. Compared to WT, *ApoE^−/−^* mice showed a significant increase in the number of RGLs labelled by GFAP (+82%) and a significant depletion of new neurons labelled by DCX (−41%), while ApoE deficiency did not significantly affect the number of TAPs labelled by Ki67 and SOX2 nor the density of mature neurons labelled by NeuN (Figure 1b). These results indicated that ApoE is important in the regulation of the neurogenetic process, and that its absence has a stimulatory effect on RGLs and an inhibitory effect towards newly generated immature neurons. These findings also suggest that ApoE functions to inhibit activation of quiescent NSCs in vivo.

ApoE modulates neuroinflammation through several pathways, and ApoE knock-out has been shown to induce inflammatory responses related to microglia in the neonatal mice brain via astrocytes [20]. To investigate the effect of ApoE deficiency on the basal level of microglia and astroglia in the hippocampus, brain sections from WT and ApoE-deficient mice of 7 months of age were immunostained for IBA1 and GFAP, labelling resting microglial and astroglial cells, respectively. Our results show that ApoE deficiency did not significantly change the number of microglial or astrocytes cells resident in the hippocampus (Figure 1c), although differences in the activation status cannot be ruled out.

### 3.2. Effects of ApoE Deficiency on the Expression of miRNAs and mRNAs Involved in the Neurogenetic Process

**Figure 2:**
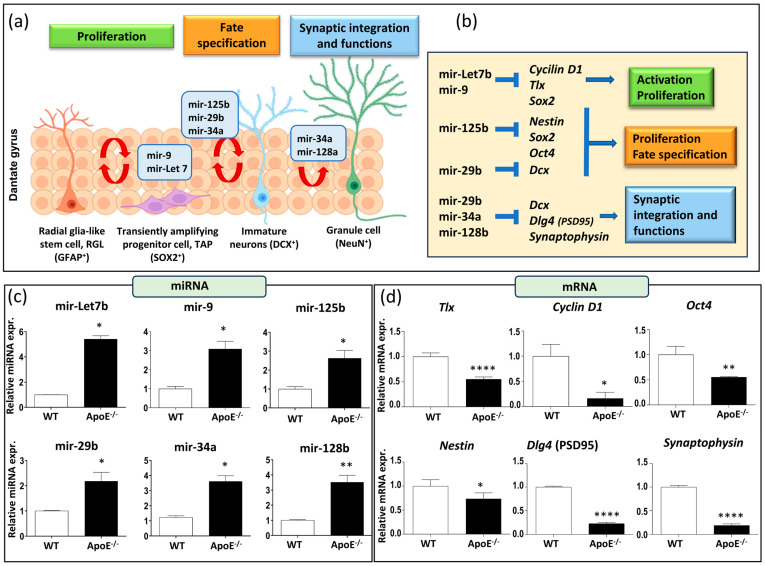
Effects of a lack of *ApoE* on miRNA and mRNA of neurogenesis-related genes. (**a**) Schematic representation of miRNAs involved in RGL cells activation, proliferation, and differentiation. (**b**) miRNAs downstream target genes. (**c**) Neurogenesis-related miRNAs expression (mir-9, mir-Let7b, mir-34a, mir-125b, mir-29b, mir-128b) in *ApoE^−/−^* and WT mice. (**d**) mRNAs expression of neurogenesis-related genes (*Tlx*, *Cyclin D1*, *Oct4*, *Nestin*, *PSD95*, *Synaptophysin*) in *ApoE^−/−^* and WT mice. Data are shown as mean ± SEM. Differences were tested with Student’s *t*-test. * *p* < 0.05; ** *p* < 0.01; **** *p* < 0.0001.

Emerging data highlight microRNAs’ crucial role in regulating various physiological processes in adult neurogenesis, including proliferation, fate determination, synaptic integration, and plasticity. They maintain the balance between stem cell self-renewal and fate determination by modulating the expression of regulatory genes in neural stem cells (Figure 2a,b). Our focus was on specific microRNA involved in the regulation of neural stem cell fate decisions (mir-9 and mir-Let7b), neural stem/progenitor cells equilibrium (mir-125b and mir-29b), and synaptic integration and functions (mir-34a and mir-128b). All these microRNA can influence specific targets, such as *Tlx* (the orphan nuclear receptor, also known as NR2E1), *Cyclin D1*, *Oct4* (Octamer-binding transcription factor 4), *Nestin*, *Dlg4* (also known as postsynaptic density protein 95, PSD95), and *synaptophysin*, as reported in Figure 2b.

It is noteworthy that our results comparing the baseline miRNA expression levels in WT and *ApoE^−/−^* mice of 7 months of age revealed a significant increase in the expression of all the miRNA tested (Figure 2c). Additionally, these increases in miRNA expression were supported by a simultaneous decrease in the expression of their principal target genes (Figure 2d). Altogether, our results suggest that a lack of *ApoE* affects not only hippocampal neurogenesis regulation but also synaptic functionality and integration.

### 3.3. Radiation-Induced Alterations in the Hippocampus of WT Mice 

**Figure 3:**
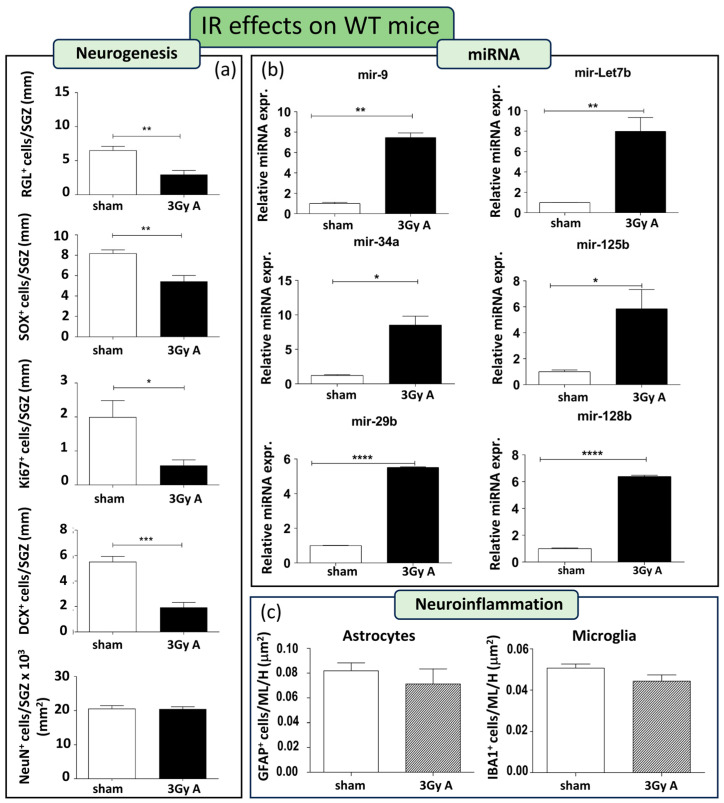
Cellular and molecular alterations in the hippocampus of WT mice at 150 days post-irradiation following 3 Gy-acute irradiation (3Gy A). (**a**) Immunostaining and relative quantification of neurogenesis markers (GFAP, SOX2, Ki67, DCX, and NeuN) in irradiated and unexposed mice. (**b**) Expression levels of neurogenesis-related miRNAs (mir-9, mir-Let7b, mir-34a, mir-125b, mir-29b, mir-128b) in irradiated and unexposed mice. (**c**) Immunostaining for astrocytes and microglia cells, labelled by GFAP or IBA1, respectively, and their relative quantification in the ML and H of the hippocampus. Data are reported as mean ± SEM * *p* < 0.05; ** *p* < 0.01; *** *p* < 0.001, **** *p* < 0.0001, for comparison with controls (Student’s *t*-test unpaired).

As a first step, we characterized radiation effects on hippocampal neurogenesis in WT mice. To this aim, WT mice were acutely irradiated with 3 Gy of X-rays at 8 weeks of age. Stage-specific adult neurogenesis markers, namely GFAP, Ki67, SOX2, DCX, and NeuN, were analyzed on consecutive sections to evaluate possible radiation-induced alterations in the cellular composition of the SGZ of the DG. In irradiated WT mice, a notable depletion was observed in almost all cell compartments of the DG. This included a 54% reduction in GFAP+ cells (*p* = 0.0042), a 34% decrease in SOX2+ cells (*p* = 0.0067), a 71% decline in Ki67+ cells (*p* = 0.018), and a substantial 65% reduction in DCX+ cells (*p* = 0.0004). Only mature neurons labeled by NeuN remained unchanged (Figure 3a). 

This indicates that acute irradiation with 3 Gy persistently impairs neurogenesis in WT mice up to 5 months post-irradiation. In addition, we examined the alterations induced by IR in the miRNAs. There is a clear and significant upregulation in all the miRNAs examined (i.e., mir-9, mir-Let7b, mir-34a, mir-125b, mir-29b, and mir-128b; Figure 3b), indicative of an involvement of these miRNAs in the response of WT mice to radiation. 

Finally, we evaluated the neuroinflammatory response 5 months after acute irradiation. Brain sections from sham controls and irradiated WT mice, were immunostained for IBA1 and GFAP labelling microglial and astroglial cells, respectively. Staining was evaluated in the molecular layer and in the hilus. The number of microglial and astroglial cells was unchanged in irradiated WT mice compared to their unirradiated controls (Figure 3c), although we cannot exclude differences in the activation status.

### 3.4. Influence of ApoE Deficiency in Radiation Response in the Hippocampus 

ApoE plays important roles in neuroprotection, and it has been shown to be required for recovery after injury in the DG [14]. To investigate whether the lack of ApoE influences radiation response in the hippocampus, we first evaluated the response of *ApoE^−/−^* and WT mice to irradiation with 3 Gy.

**Figure 4:**
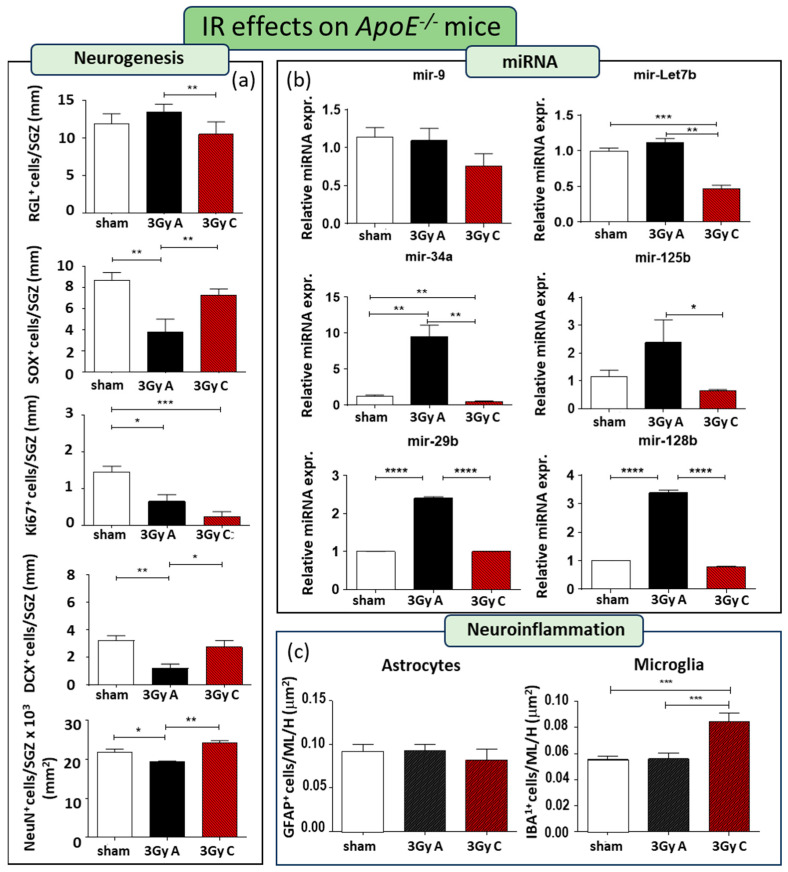
Cellular and molecular alterations in the hippocampus of *ApoE^−/−^* mice at 150 days after acute irradiation (3 Gy A) or after 150 days of chronic irradiation (total dose of 3 Gy C). (**a**) Immunostaining and relative quantification for neurogenesis-related markers (GFAP, SOX2, Ki67, DCX, and NeuN). (**b**) Relative expression of neurogenesis-related miRNAs (mir-9, mir-Let7b, mir-34a, mir- 125b, mir-29b, mir-128b). (**c**) Immunostaining for astrocytes and microglia cells, labelled by GFAP or IBA1, respectively, and their relative quantification in the ML and H of the hippocampus. Data are reported as mean ± SEM * *p* < 0.05; ** *p* < 0.01; *** *p* < 0.001; **** *p* < 0.0001 for comparison with controls (Student’s *t*-test unpaired).

A single acute dose of 3 Gy of X-rays resulted in impaired neurogenesis, leading to a 56% reduction in SOX2+ cells (*p* = 0.0067), a 54% decrease in Ki67+ cells (*p* = 0.01), a 69% decline in DCX+ cells (*p* = 0.0011), and an 11% decrease in NeuN+ cells (*p* = 0.036) (Figure 4a). However, comparing radiation responses between WT and *ApoE^−/−^* mice, we detected a few differences, mainly consisting of a lack of reduction of GFAP-labelled RGL cells and a reduction in the number of NeuN mature granule neurons in *ApoE^−/−^* mice, suggesting that a lack of *ApoE* persistently affects DG recovery after radiation-induced injury (Figure 4a). The effects of this single irradiation on the miRNAs expression significantly induce upregulation of mir-34a, mir 29b, and mir-128b, suggesting their role not only in the regulation of synaptic functions but also in their contribution on early stage of neurogenesis (Figure 4b). 

Additionally, to address the importance of dose-rate on the effects on adult neurogenesis, we compared the effects induced by a cumulative dose of 3 Gy administered acutely (at a rate of 0.89 Gy/min) or chronically at a lowdose-rate (20 mGy/day over 150 days) in *ApoE^−/−^* mice. Compared to unirradiated *ApoE^−/−^* mice, chronic irradiation only caused a significant decrease of 84% Ki67+ cells (*p* = 0.0008), without modifying the fraction of TAP cells labelled by (SOX2+), and newborn (DCX+) and mature neurons (NeuN+), indicating that chronic low dose-rate exposure constitutes a lower risk factor for the perturbation of hippocampal neurogenesis (Figure 4a). Moreover, chronic irradiation primarily affects mir-Let7b and mir34a, which exhibited significant downregulation compared to both irradiated and unexposed control groups (Figure 4b), consistent with the minor impact on neurogenesis.

Immunostaining for microglial and astroglial cells indicated no alterations in these two cell populations when mice were acutely irradiated, consistent with observations in WT mice (Figure 4c). However, our results demonstrate that when a low dose rate was applied, with irradiation consequently extended over 5 months, a significantly higher density of microglial cells were observed in the hippocampus compared to unirradiated and acutely irradiated hippocampi, showing a long-term microglia-mediated neuroinflammation (Figure 4c).

## 4. Discussion

In this work, we used the *ApoE^−/−^* mouse model to investigate the potential role of the *ApoE* gene in the modulation of the neurogenesis process and neuroinflammation within the hippocampus and in response to acute and chronic irradiation. A comprehensive approach was employed to gain a mechanistic understanding of the miRNA-mediated transcriptional consequences of a lack of ApoE on gene regulation within the neurogenic program in the hippocampus during homeostasis and in response to irradiation.

### 4.1. Effects of ApoE Deficiency in the Hippocampus

The hippocampus is involved in the consolidation of information from short-term to long-term memory, as well as learning and spatial memory. ApoE primarily functions as a lipid transport protein and a lipid and cholesterol regulator. It represents a major cholesterol carrier in the brain, playing a crucial role in membrane repair, hippocampal neurogenesis, and synaptic plasticity [21]. 

Our results show that the ApoE deficiency perturbs hippocampal neurogenesis by enlarging the pool of the hippocampal staminal cells (RGLs) and decreasing the number of DCX-expressing newborn neurons. This expansion of the RGL cells pool is supported by the reported increase in the RGLs’ proliferation potential in *ApoE*-deficient mice [21]. It is interesting to note that *ApoE* expression in the normal DG of the hippocampus is localized to the GFAP-expressing processes of early progenitors and that progenitor cells derived from *ApoE*-deficient mice exhibit an increased self-renewal potential, forming nearly five times more neurospheres than progenitors from WT mice [21,22].

Accumulating evidence suggests that miRNAs, a class of post-transcriptional gene expression regulators, play a crucial role in the regulatory mechanisms operating in adult neurogenesis [23,24]. A major focus of the present work was to understand whether alterations in the expression of certain pivotal miRNAs could help to clarify the role of *ApoE* in the regulation of neurogenesis. A selected set of miRNAs, such as mir-9 and mir-Let7b for activation/proliferation, mir-125b and mir-29b for proliferation/fate specification, and mir-34a and mir-128b for synaptic integration and functions, has been utilized to help in the analysis of key stages in neurogenesis [25,26,27,28,29,30,31], even though this miRNAs panel represents an oversimplification of the intricate miRNA network governing the life of neurons, spanning from specification and differentiation to integration into existing neuronal networks. Moreover, we identified selected mRNAs involved in different neurogenesis-related processes and that are direct targets of the miRNAs tested.

Compared to WT mice, we demonstrated a significant upregulation of all the miRNAs tested in the hippocampus of *ApoE^−/−^* mice, including mir-Let7b, mir-9, mir-25b, mir-29b, mir-34a, and mir-128b, that correlated with a significant decrease in the expression of matching target mRNAs (*Tlx*, *Cyclin D1*, *Oct4*, *Nestin*, *Dlg4* (*PSD95*), *and Synaptophysins*). 

Specifically, the upregulation of mir-Let7b and mir-9 levels matches with the downregulation of *Cyclin D1* and *Tlx* mRNAs expression [27,28,29]. Furthermore, the increase of mir-29b and mir-125b levels may potentially contribute to the observed reduction in the number of DCX-expressing cells within the hippocampal dentate gyrus [25,26]. Mir-29a was reported to modulate axon branching by targeting DCX in primary neurons [26]. Moreover, it is known that mir-34a acutely regulates synaptic efficacy in the adult DG in vivo [32], and that mir-128b is implied in the control of neurogenesis and cooperates in the regulation of dendritic arborization [33,34]. The upregulation of these miRNA in the *ApoE*-deficient mouse model, could be important in the regulation of synaptic functions and integration. Importantly, we demonstrated here that in mice lacking *ApoE*, synaptogenesis is severely impaired as shown by the strong reduction of the expression of the synaptic markers *PSD95* and *synaptophysin*. it is noteworthy that previous works suggested a synaptic reduction in *ApoE^−/−^* vs. WT mice [22,35]. Interestingly, age-dependent synaptic loss and learning deficits have previously been detected in *ApoE*-deficient mice [13]. Furthermore, recently, astrocytic *ApoE* deficiency has been shown to result in a simplification of dendritic tree structure and reduction in spine density in DG neurons associated with spatial memory deficit [36].

In this study, we also demonstrated that neurogenesis deficits in *ApoE* null mice are not accompanied by significant alterations in the neuroinflammation state, since the number of hippocampal microglial or astroglial cells was unchanged. Enhanced IBA1 protein expression in *ApoE*-deficient mice was recently reported but restricted to the neonatal period and fully restored at 6 months of age [22]. Therefore, our histochemical analysis in 7-month-old mice aligned with the reported literature. 

### 4.2. Role of ApoE in Radiation Response

We here used *ApoE^−/−^* mice, a model related to atherosclerosis, Alzheimer’s disease and other vascular diseases, and CNS dysfunction [4,5,10,11,37], and exposed them to a dose of 3 Gy [38], which is relevant to cancer therapy. This dose was given acutely, or protracted over 150 days, to gain insights into the effects of low dose and/or chronic radiation exposures on the hippocampus. 

Although the effects of ApoE in the brain are still being elucidated, ApoE has been shown to play an important role in the response to environmental challenges, including neuronal repair following injury. In our investigations, we compared irradiation effects in WT and *ApoE^−/−^* mice to elucidate the role of the *ApoE* gene in the radiation response.

In agreement with the well-known long-term consequences of irradiation on neurogenesis, involving quiescent, dividing, and differentiating neuronal populations [39,40,41], we show that compared to unexposed mice, acute irradiation of WT mice inhibits all stages of adult neurogenesis in the DG, except mature neurons. 

The first difference between WT and *ApoE^−/−^* mice when exposed to acute irradiation, is that *ApoE^−/−^* mice do not show an inhibition in the RGL stem cell pool but they show a reduction in mature neurons. This lack of blockage in the RGL stem cell pool suggests that the absence of ApoE may help protect the hippocampal stem cell compartment from radiation damage, with important implications for brain protection. 

Moreover, acute irradiation in WT mice led to a significant increase in all tested miRNAs, suggesting impairment in proliferation, fate determination, and synaptic plasticity. In contrast, we observed the absence of upregulation of mir-9 and mir-Let7b in irradiated *ApoE^−/−^* mice. It has been reported that mir-9 is expressed in neural stem cells and upregulated upon neural differentiation [24] while overexpression of mir-Let7b causes reduced proliferation and an increase in neural differentiation. Since mir-9 and mir-Let7b are implicated in the activation and proliferation of stem cells, their unchanged levels may correlate with the preservation of the RGL cell population. 

Our data also show that while a lack of *ApoE* caused a basal upregulation of all tested miRNAs, acute irradiation of these mice only caused a further significant upregulation of synaptic functional-related miRNAs (mir-29b, mir-34a, and mir-128b). Overall, our results indicate that irradiation perturbs the network regulating synaptogenesis in the hippocampus and that this deregulation is exacerbated in the *ApoE* null genotype.

Data from the literature showing that irradiation modifies the expression of synaptic proteins, such as PSD95, support our findings, although discrepant data are reported. While PSD95 expression was reported to increase in a mouse model after irradiation with γ-rays (1 and 10 Gy) [42] or 250 MeV protons [43], other groups found that the PSD95 expression was not altered after irradiation with high doses of X-rays [44] or even decreased in neurons after X-irradiation with 0.5 or 1 Gy [45]. 

The rate at which radiation is delivered influences its impact and the biological response, also having strong implications for radiation protection. Thus, we here investigated the effect of acute or chronic radiation exposure on the hippocampus of *ApoE* null mice. Hippocampal neurogenesis was less impacted by chronic irradiation in *ApoE^−/−^* mice when compared to acute irradiation, with a unique deficit in the number of Ki67 positive proliferating cells. A possible explanation could be that the extremely low dose rate (20 mGy/day, equivalent to 15 μGy/min) employed in chronic irradiation might not induce cell death but cell cycle arrest, providing an opportunity for DNA damage repair processes. In addition, compared to acute, chronic irradiation also has a lower impact on miRNAs deregulation, with only mir-Let7b and mir-34a significantly downregulated. Overall, in the hippocampus of *ApoE* mice, chronic irradiation generally resulted in fewer consequences compared to acute exposure, except for microglia-mediated neuroinflammation that was not observed at 150 days after acute irradiation. It is noteworthy that the significant increase in microglial cells after chronic irradiation of *ApoE^−/−^* mice coupled with a significant decrease in mir-Let7b, which, among others, has a regulatory role in neuroinflammatory processes [46]. The role of mir-Let7b in inflammation is well documented and it has been reported to modulate inflammation by targeting cytokines such as IL-6 and IL-10, as well as the receptor TLR4 [47]. 

In summary, our data indicate that *ApoE* deficiency leads to impaired adult hippocampal neurogenesis, marked by elevated miRNA levels and reduced expression of neurogenesis-related genes involved in key stages like activation, proliferation, fate specification, and synaptic function. Interestingly, there is no impact on neuroinflammation. When compared to 3-Gy-irradiated WT mice, similarly, treated *ApoE^−/−^* mice show unaffected RGLs but inhibition of mature neurons. Mechanistically, while all the tested miRNAs were upregulated in WT mice following irradiation, *ApoE* null mice exhibited upregulation limited to miRNAs associated with synaptogenesis. In *ApoE^−/−^* mice, chronic irradiation induced milder effects on neurogenic processes, inhibiting the proliferating neurogenic cell compartment and mir-Let7b and mir-34a, with detected microglia-related neuroinflammation. 

## 5. Conclusions

In conclusion, our study highlights the importance of ApoE in hippocampus homeostasis and in mediating the response to both acute and chronic low dose-rate irradiation. Current evidence underscores ApoE’s role in maintaining physiological brain homeostasis. Ongoing research is investigating experimental therapeutic approaches targeting ApoE, aiming for preventive or corrective outcomes in neurodegenerative proteinopathies, particularly AD [48]. However, before manipulating ApoE levels as a safe therapeutic intervention to prevent or delay AD related dementias, a comprehensive understanding of the prolonged repercussions of *ApoE* loss of function in critical brain regions, such as the hippocampus, is crucial.

In addition, understanding the role of ApoE in an ionizing radiation exposure scenario may shed light on potential mechanisms underlying radiation-induced neurocognitive effects and may offer insights into therapeutic strategies for mitigating radiation-induced brain injury. Further research in this area is of high translational relevance to human health and it is warranted to fully elucidate the intricate interplay between ApoE and radiation exposure in the brain.

## Figures and Tables

**Table 1:** Summary of the experiment mice groups with age at irradiation and sacrifice.

WT				*ApoE^−/−^*			
	Age at IR (Weeks)	No. of Mice	Age at Sacrifice (Months)		Age at IR (Weeks)	No. of Mice	Age at Sacrifice (Months)
Sham	8	24	7	Sham	8	24	7
3 Gy A	8	24	7	3 Gy A	8	24	7
				3 Gy C	8	24	7

A: acute irradiation; C: chronic irradiation.

**Table 2:** Primers for real-time qPCR.

Mouse Gene	Forward Primer	Reverse Primer
***Tlx***	5′-CGATTAGACGCCACTGAA-3′	5′-GGTATCTGGTATGAATGTAGC-3′
***Cyclin D***	5′GCAAGCATGCACAGACCTT-3′	5′-GTTGTGCGGTAGCAGGAGA-3′
***Oct4***	5′-AAAGCCCTGCAGAAGGAGCTAGAA -3′	5’-AACACCTTTCCAAAGAGAACGCCC -3′
***Nestin***	5′-AGGCTGAGAACTCTCGCTTG-3′	5′-TGAGAAGGATGTTGGGCTGA-3′
***Dlg4* (*PSD95*)**	5′-CTTCATCCTTGCTGGGGGTC-3′	5′-TTGCGGAGGTCAACACCATT-3′
***Synaptophysin***	5′-CTGCGTTAAAGGGGGCACTA-3′	5′-GGGATTTCCATTGATGACAAG-3′
***Gadph***	5′-CATGGCCTTCCGTGTTCCTA -3′	5′-GCGGCACGTCAGATCCA -3′

## Data Availability

The original contributions presented in the study are included in the article, further inquiries can be directed to the corresponding author/s.
